# A mosaic pathogenic variant in *MSH6* causes MSH6-deficient colorectal and endometrial cancer in a patient classified as suspected Lynch syndrome: a case report

**DOI:** 10.1007/s10689-023-00337-0

**Published:** 2023-06-15

**Authors:** Romy Walker, Mark Clendenning, Jihoon E. Joo, Jessie Xue, Khalid Mahmood, Peter Georgeson, Julia Como, Sharelle Joseland, Susan G. Preston, James M. Chan, Mark A. Jenkins, Christophe Rosty, Finlay A. Macrae, Stephanie Di Palma, Ainsley Campbell, Ingrid M. Winship, Daniel D. Buchanan

**Affiliations:** 1 Colorectal Oncogenomics Group, Department of Clinical Pathology, The University of Melbourne, 305 Grattan Street, Parkville, VIC 3010 Australia; 2 University of Melbourne Centre for Cancer Research, Victorian Comprehensive Cancer Centre, 305 Grattan Street, Parkville, VIC 3010 Australia; 3 Melbourne Bioinformatics, The University of Melbourne, Melbourne, Parkville, VIC 3010 Australia; 4 Centre for Epidemiology and Biostatistics, The University of Melbourne, Parkville, VIC 3010 Australia; 5 Envoi Specialist Pathologists, Brisbane, QLD 4059 Australia; 6 University of Queensland, Brisbane, QLD 4072 Australia; 7 Genomic Medicine and Familial Cancer Centre, The Royal Melbourne Hospital, Parkville, VIC 3000 Australia; 8 Colorectal Medicine and Genetics, The Royal Melbourne Hospital, Parkville, VIC 3000 Australia; 9 Department of Medicine, The University of Melbourne, Melbourne, VIC 3000 Australia; 10 Clinical Genetics Unit, Austin Health, Melbourne, VIC 3084 Australia

**Keywords:** DNA mismatch repair, *MSH6*, Mosaicism, Suspected Lynch syndrome, Targeted tumor sequencing, Droplet digital PCR

## Abstract

Germline pathogenic variants in the DNA mismatch repair (MMR) genes (Lynch syndrome) predispose to colorectal (CRC) and endometrial (EC) cancer. However, mosaic variants in the MMR genes have been rarely described. We identified a likely *de novo* mosaic *MSH6*:c.1135_1139del p.Arg379* pathogenic variant in a patient diagnosed with suspected Lynch syndrome/Lynch-like syndrome. The patient developed MSH6-deficient EC and CRC at 54 and 58 years of age, respectively, without a detectable germline MMR pathogenic variant. Multigene panel sequencing of tumor and blood-derived DNA identified an *MSH6* somatic mutation (*MSH6*:c.1135_1139del p.Arg379*) common to both the EC and CRC, raising suspicion of mosaicism. A droplet digital polymerase chain reaction (ddPCR) assay detected the *MSH6* variant at 5.34% frequency in normal colonic tissue, 3.49% in saliva and 1.64% in blood DNA, demonstrating the presence of the *MSH6* variant in all three germ layers. This study highlights the utility of tumor sequencing to guide sensitive ddPCR testing to detect low-level mosaicism in the MMR genes. Further investigation of the prevalence of MMR mosaicism is needed to inform routine diagnostic approaches and genetic counselling.

## Introduction

Lynch syndrome is caused by germline pathogenic variants in one of the DNA mismatch repair (MMR) genes where carriers have an increased risk of developing colorectal (CRC) and endometrial (EC) cancer, among other cancers. Mosaicism of hereditary CRC genes is not uncommon [1], but mosaicism in the MMR genes is thought to be rare with only a few cases reported to date (Table 1) [2-5]. Here, we report the first case of a mosaic *MSH6* gene pathogenic variant in an EC- and CRC-affected individual diagnosed with suspected Lynch syndrome.

**Table 1:** Characteristics of individuals reported in the literature and from this study identified to have a DNA mismatch repair gene mosaic pathogenic variant

#	Gene	Variant	Type of Mosaic Mutation	Transmitted to Offspring	Sex	Personal Cancer History	MSI Status	Pattern of MMR Protein Loss of Expression by IHC	Reference
1	*MLH1*	c.1050del p.(Gly351Aspfs*16)	revertant mosaicism (reversion of inherited mutation)	Possible but no biological offspring	F	Synchronous EC and ovarian cancer @44, CRC @48	NT *	NT	Pastrello et al. [3]
2	*MSH2*	c.2541del p.(Ala848Profs*44)	*de novo*	Yes, son CRC @54	F	CRC @79	MSI-H	MSH2	Sourrouille et al. [2]
3	*MLH1*	c.518_519del p.(Tyr173Trpfs*18)	*de novo*	Possible but no biological offspring	M	CRC @31	MSI-H	MLH1/ PMS2	Geurts-Giele et al. [4]
4	*MSH2*	c.1269del, p.(Lys423Asnfs*15)	likely *de novo*	Yes, daughter unaffected	F	EC @45, CRC @60	NT (EC), MSI-H (CRC)	MSH2/ MSH6 (EC), MSH2/ MSH6 (CRC)	Guillerm et al. [5]
5	*MSH6*	c.1135_1139del p.Arg379*	likely *de novo*	Possible but no biological offspring	F	EC @55, CRC @57	MSI-H	MSH6 (EC), MSH6 (CRC)	This study

*CRC* colorectal cancer; *EC* endometrial cancer; *MSI-H* high levels of microsatellite instability;* MMR* DNA mismatch repair; *IHC* immunohistochemistry;* NT* not tested

*Tumor not-amplifiable

## Case Presentation

**Fig. 1:**
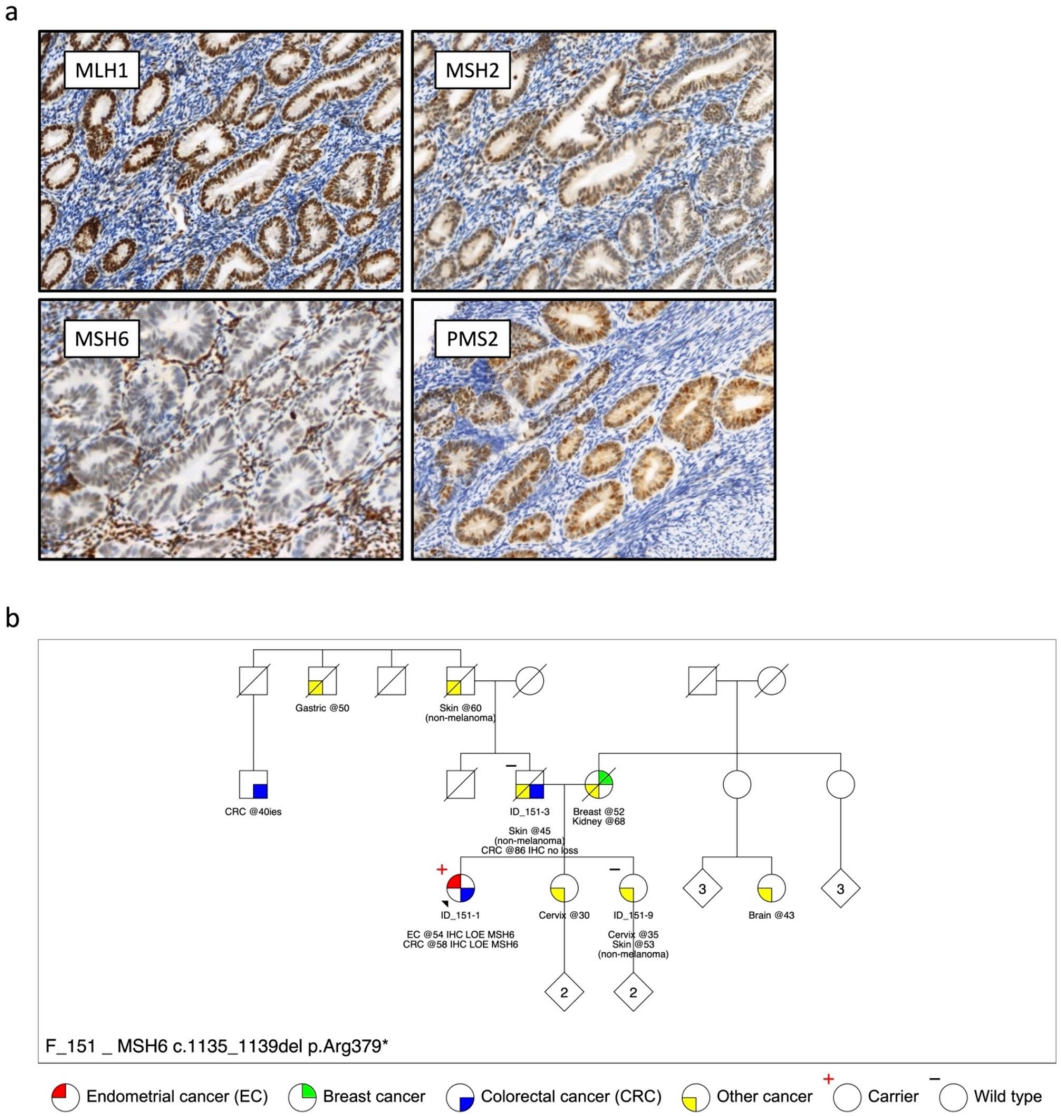
**a** DNA mismatch repair immunohistochemical staining of the endometrial tumor showing loss of MSH6 protein expression and retained expression of MLH1, MSH2 and PMS2 proteins. **b** Display of the family pedigree. The proband (ID_151-1) is indicated by the black arrow. The carrier of the *MSH6*:c.1135_1139del p.Arg379* pathogenic variant is indicated with a red plus symbol and the two additional family members who were tested were non-carriers indicated with a black minus symbol

The patient (ID_151-1) developed EC at the age of 54 and underwent a total hysterectomy and bilateral salpingo-oophorectomy. The cancer was a well-differentiated (FIGO grade 1) endometrioid adenocarcinoma showing superficial myometrial invasion. There was no evidence of cervical or adnexal involvement. MMR immunohistochemical staining of the tumor showed solitary loss of MSH6 protein expression (Fig. 1a). In December 2016, the patient was referred to a family cancer clinic where germline MMR gene testing was completed by next-generation sequencing (NGS) and multiplex ligation-dependent probe amplification. No clinically actionable germline pathogenic variants in *MSH6* or the other MMR genes were identified. Patients with tumor MMR-deficiency without evidence of a germline pathogenic variant or tumor *MLH1* methylation are given a diagnosis of suspected Lynch syndrome/Lynch-like syndrome, also referred to as unexplained tumor MMR-deficiency, therefore, the patient (ID_151-1) was diagnosed with suspected Lynch syndrome.

In September 2017, the patient developed a second primary cancer within the caecum at the age of 58. A right hemicolectomy was performed to remove a stage IIA high-grade mucinous carcinoma that demonstrated solitary loss of MSH6 protein expression by MMR immunohistochemistry. The personal and family cancer histories are shown in Fig. 1b. The patient had no children. She was referred from the clinic to the ANGELS study (Applying Novel Genomic approaches to Early-onset and suspected Lynch Syndrome colorectal and endometrial cancers) for tumor sequencing [6]. The study was approved by the University of Melbourne human research ethics committee (HREC#1750748) and the institutional review boards at the Austin Health Clinical Genetics Service. All participants in this study signed an ethics-approved consent form.

## Investigations

**Fig. 2:**
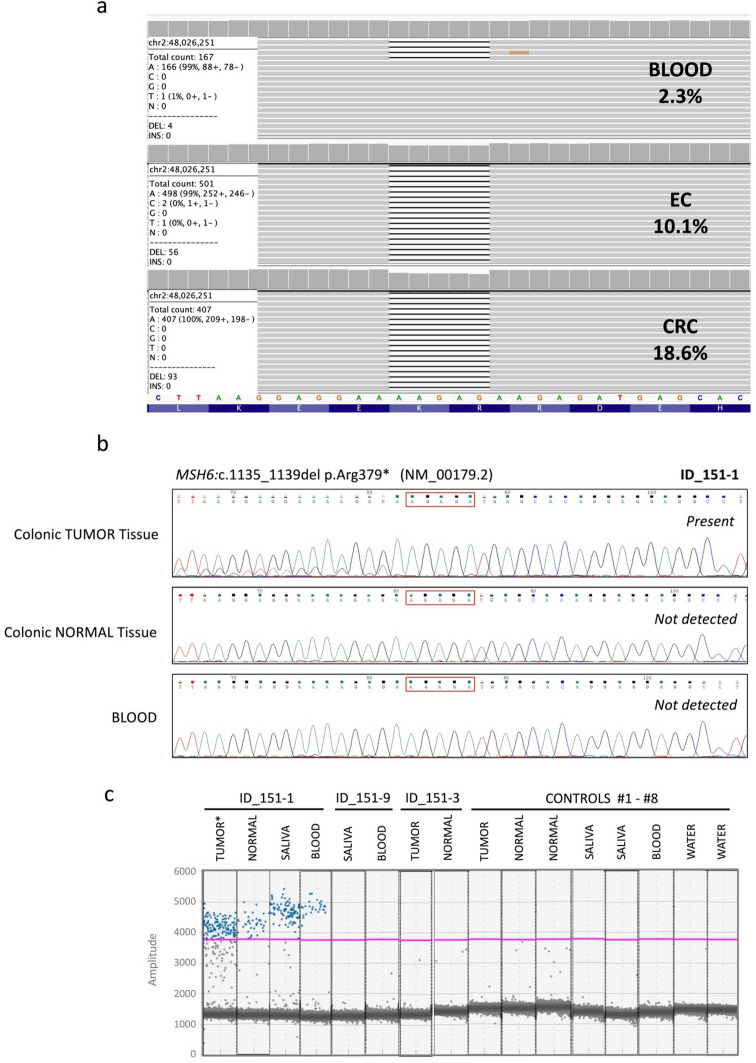
**a** Integrative Genomics Viewer display of the *MSH6*:c.1135_1139del p.Arg379* pathogenic variant and its read depth in the patient’s blood, endometrial cancer and colorectal cancer DNA from targeted multigene panel sequencing. **b** Sanger sequencing analysis of the probands (ID_151-1) colorectal cancer tumor tissue, colonic normal tissue and blood, showing the presence of the *MSH6*:c.1135_1139del p.Arg379* variant in the tumor tissue but not detectable in the colonic normal or blood-derived DNA. **c** Results from the droplet digital polymerase chain reaction (ddPCR) assay displaying the abundance of the detected *MSH6*:c.1135_1139del p.Arg379* variant in different tissue DNA samples from the patient (ID_151-1), but not in the father (ID_151-3), sister (ID_151-9) or controls. The purple line indicates a manually placed threshold. *Only the colorectal tumor tissue was tested

The patient’s (ID_151-1) EC and CRC tumor tissue DNA and matched blood-derived DNA were tested on a custom-designed multigene panel. Details of the panel sequencing assay and bioinformatic pipeline have been published previously [6]. The mean on-target coverage for the EC, CRC and blood-derived DNA were 489x, 927x and 69x, respectively. The genomic calculated tumor cellularity for the EC and CRC were 21% and 37%, respectively. MANTIS determined both the EC and CRC to be MSI-H, with scores of 0.22 and 0.49 respectively (> 0.16 = MSI-H) [7]. Panel sequencing identified a single *MSH6* somatic mutation (NM_000179.2: c.1135_1139del p.Arg379*) at a variant allele frequency (VAF) of 10.1% in the EC and two *MSH6* somatic mutations (c.3261del p.Phe1088Serfs*2 and c.1135_1139del p.Arg379*) in the CRC at VAFs of 23% and 18.6%, respectively. The *MSH6*:c.1135_1139del p.Arg379* mutation, common to both tumors, had a VAF of 2.3% in the matched blood-derived DNA (Table 2; Fig. 2a). No other variants were in common between the EC and CRC. These results suggested the *MSH6*:c.1135_1139del p.Arg379* mutation was potentially mosaic in at least two germ layers. To exclude the possibility of a sequencing artefact, Sanger sequencing confirmed the presence of the *MSH6* mutation in the CRC tumor but did not detect the variant in the normal non-adjacent colonic mucosa or blood DNA samples (Fig. 2b; Table 2).

**Table 2:** The variant allele frequency results from testing of the *MSH6*:c.1135_1139del p.Arg379* variant in different tissue sources from the proband and relatives using next-generation sequencing (NGS), Sanger sequencing (Sanger) and digital droplet polymerase chain reaction (ddPCR) methodologies

	Patient type	Patient ID	Germ layer	DNA source	NGS	Sanger^*^	ddPCR^**^
TEST	Proband	ID_151-1	Ectoderm	CRC tumor	18.6%	Present	20.2%
		ID_151-1	Ectoderm	EC tumor	10.1%	NT	NT
		ID_151-1	Ectoderm	Normal colonic mucosa	NT	Not detected	5.3%
		ID_151-1	Mesoderm	Saliva	NT	NT	3.5%
		ID_151-1	Endoderm	Blood	2.3%	Not detected	1.6%
	Sister	ID_151-9	Mesoderm	Saliva	NT	NT	0%
		ID_151-9	Endoderm	Blood	NT	NT	0%
	Father	ID_151-3	Ectoderm	CRC tumor	NT	NT	0%
		ID_151-3	Ectoderm	Normal colonic mucosa	NT	NT	0%
CONTROL	Control #1	ID_409	Ectoderm	CRC tumor	NT	NT	0%
	Control #2	ID_082	Ectoderm	Normal colonic mucosa	NT	NT	0%
	Control #3	ID_436	Ectoderm	Normal colonic mucosa	NT	NT	0%
	Control #4	ID_236	Mesoderm	Saliva	NT	NT	0%
	Control #5	ID_007	Mesoderm	Saliva	NT	NT	0%
	Control #6	ID_398	Endoderm	Blood	NT	NT	0%
	Control #7	ID_001	NA	No template control	NT	NT	0%
	Control #8	ID_002	NA	No template control	NT	NT	0%

Abbreviations: ID, identification number; CRC, colorectal cancer; EC, endometrial cancer; NGS, next-generation sequencing; Sanger, Sanger sequencing; ddPCR, digital droplet polymerase chain reaction; NA, not applicable; NT, not tested

* Primers used for Sanger sequencing: Forward 5'-TAGTGGAGGTGGTGATGACAGTAGT'3', Reverse 5'- CTCATCCCAGGAGTACAAGAATTGA-3'

** Primers used for digital droplet polymerase chain reaction: Forward 5'-AGTAGTCGCCCTACTGTTT-3', Reverse 5'-TCAGGCACATAGAGTGTAGAT-3'

The *MSH6*:c.1135_1139del p.Arg379* variant, confirmed as pathogenic in ClinVar and InSiGHT databases, was tested across different germ layer DNA samples from the proband (ID_151-1), father (ID_151-3), sister (ID_151-9) and unrelated controls using a customized ultra-sensitive droplet digital polymerase chain reaction (ddPCR) assay (Table 2). The *MSH6* variant was detected at low levels in the normal colonic mucosa (5.3% VAF), saliva (3.5% VAF) and blood (1.6% VAF) DNA from the patient but in none of the controls (#1-#8) (Table 2; Fig. 2c), confirming mosaicism in all three germ layers thus suggesting an early embryonic event post zygosis. The MMR-proficient CRC tissue and non-adjacent normal colonic tissue from the father (ID_151-3) and blood and saliva DNA from the sister (ID_151-9) did not show evidence of the *MSH6* variant by ddPCR (Table 2). The mother was deceased prior to study recruitment and could not be screened for the *MSH6* variant.

## Discussion

Currently, MMR mosaicism appears to be rare with only a handful of cases reported to date [2–5] (Table 1). The *MSH6*:c.1135_1139del p.Arg379* variant is the first report of a mosaic pathogenic variant in the *MSH6* gene and was identified as a somatic mutation in both the EC and CRC following panel testing in a woman presenting with MSH6-deficiency in both her tumors. ddPCR of the *MSH6* variant enabled confirmation of mosaicism demonstrating the variant at low-levels in multiple tissue samples encompassing the endoderm (colon), ectoderm (saliva) and mesoderm (blood). This suggests the variant occurred early in embryogenic development and is potentially present in the primordial germ cells. The *MSH6* pathogenic variant may therefore be heritable. Since the patient did not have and can no longer have biological offspring, we did not test for gonadal mosaicism but preimplantation genetic testing may be recommended where a patient is planning to have children, since detection of a mosaic pathogenic MMR variant in gonadal cells would increase the risk of cancer for all carrier children. The patient (ID_151-1), now diagnosed with mosaic Lynch syndrome, can undergo risk-appropriate clinical management while the father and sister, who had no evidence of the *MSH6* variant in their DNA samples, can now be confirmed as non-carriers and are released from intensive screening surveillance.

This case of an *MSH6* mosaic variant was identified in a person with a diagnosis of suspected Lynch syndrome/Lynch-like syndrome. Tumor testing of suspected Lynch syndrome cases has shown that the predominant etiology is two somatic MMR mutations causing biallelic MMR gene inactivation [5]. When considering who to screen for MMR mosaic variants, cases with somatic MMR mutations in the absence of a germline MMR pathogenic variant are the ideal candidates. Of the MMR mosaic cases identified to date, 3/5 developed multiple Lynch syndrome spectrum cancers (Table 1) raising the suspicion of an undetected germline pathogenic variant. It is likely that the presentation of these MMR mosaicism cases with multiple cancers is a bias of cases selected for mosaicism testing. The ability to test multiple tumors for somatic MMR mutations, both with loss of MSH6 expression, enabled us to target the ddPCR screening to a single variant shared between the tumors. A common MMR mutation in multiple tumors from the same patient may also be indicative of a primary and metastatic lesion, although more than one somatic mutation in common would support this rather than mosaicism. Testing of multiple adenomas to identify a common somatic mutation via the “adenoma first” approach, has been successfully used to identify *APC* mosaic variants in adenomatous polyposis [8]. This approach has shown *APC* mosaicism to be a more common mechanism than previously thought in unexplained adenomatous polyposis [1]. Guillerm et al. [5] and Lucia Jansen et al. [1] have both proposed decision tree models for triaging individuals diagnosed with suspected Lynch syndrome for identifying MMR gene mosaicism in patients and first-degree relatives, albeit for the research setting.

This study highlights the importance of screening for mosaicism in patients with a diagnosis of suspected Lynch syndrome and somatic MMR mutations in their tumors. Consistent with literature, this study has shown that Sanger sequencing is not sensitive enough to reliably detect low level variants [9] and alternate, more sensitive methods are required to screen for mosaic variants. The stepwise approach of MMR gene sequencing using NGS methodology in MMR-deficient tumors followed by sensitive ddPCR testing of a specific variant in DNA from multiple tissue sources from different germ layers is a recommended approach moving forward. As tumor screening and sensitive methods such as ddPCR become more widely adopted, the prevalence of MMR mosaicism may also be shown to be higher. As the true prevalence of MMR mosaicism becomes known, improvements to the diagnostic workflow can enable efficient and cost-effective screening approaches to detect all cases of Lynch syndrome, including those with mosaicism.

## Data Availability

The anonymized data analyzed during the current study is available from the corresponding author on reasonable request.
